# Socio-cultural and economic factors influencing adolescents’ resilience against the threat of teenage pregnancy: a cross-sectional survey in Accra, Ghana

**DOI:** 10.1186/s12978-015-0113-9

**Published:** 2015-12-23

**Authors:** Collins K Ahorlu, Constanze Pfeiffer, Brigit Obrist

**Affiliations:** Noguchi Memorial Institute for Medical Research, College of Health Sciences, University of Ghana, P.O Box LG581, Legon, Ghana; Swiss Tropical and Public Health Institute, Basel, Switzerland; University of Basel, Basel, Switzerland; Institute of Social Anthropology, University of Basel, Basel, Switzerland

**Keywords:** Resilience, Adolescents, Teenage pregnancy, Ghana, Sexual and reproductive health, Multi-layered social resilience framework

## Abstract

**Background:**

Adolescent pregnancy exposes female adolescents to medical, social and economic risks. In Ghana, adolescent mothers are more likely to experience complications during pregnancy and delivery as compared to older mothers. This study examined the competencies of adolescent girls to either proactively prevent teenage pregnancy or reactively cope effectively with it.

**Methods:**

A cross-sectional survey approach was used to interview 820 adolescent girls aged 15–19 years in Accra, Ghana. The main focus of the study was to examine how social capital (various kinds of valued relations with significant others), economic capital (command over economic resources, mainly cash and assets), cultural capital (personal dispositions and habits; knowledge and tradition stored in material forms and institutionalized) and symbolic capital (honour, recognition and prestige) contribute to the development of competencies of adolescents to deal with the threat of teenage pregnancy and childbirth.

**Results:**

Out of 820 adolescents interviewed, 128 (16 %) were pregnant or mothers. Adolescents in both groups (62 % never pregnant girls and 68 % pregnant/young mothers) have access to social support, especially from their parents. Parents are taking the place of aunts and grandmothers in providing sexual education to their adolescent girls due to changing social structures where extended families no longer reside together in most cases. More (79 %) pregnant girls and young mothers compared to never pregnant girls (38 %) have access to economic support (*P* = <0.001). Access to social, economic and cultural capitals was associated with high competence to either prevent or deal with pregnancy among adolescent girls.

**Conclusion:**

Findings showed that adolescent girls, especially those that get pregnant should not be viewed as weak and vulnerable because many of them have developed competencies to cope with pregnancy and childbirth effectively. Thus, focusing on developing the competencies of girls to access social, economic and cultural capitals may be an effective way of tackling the threat of teenage pregnancy than focusing only on their vulnerability and associated risks.

## Background

Young people belong to one of the biggest age groups with more than half of the world population being less than 25 years old [1]. Some 1.2 billion people, one person in five is an adolescent, ranging from 10 to 19 years [1]. The period of adolescence is a transitional one in which an individual goes through a lot of physical, emotional, psychological, cognitive and social changes [2, 3]. Individuals are expected at this stage of life to acquire and consolidate skills, attitudes and principles that are needed to prepare them for adulthood [2]. Hence, the choices one makes based on the life chances available to her/him at this critical period of life are of great importance for healthy living in adulthood. Hereby the importance of sexual and reproductive health decisions cannot be overemphasized as adolescents are considered to be the most vulnerable to sexual and reproductive health risks [4–6]. The World Health Organization (WHO) defined adolescence as all persons aged 10 to 19 years. This is further divided into younger adolescents – 10 to 14 years and older adolescents – 15 to 19 years [7].

In 2008, 11 % of all births worldwide were by adolescents aged 15–19 years with about 95 % of them occurring in low and middle income countries [8]. In Ghana, adolescent pregnancy contributes about 9 % to maternal mortality and like elsewhere in sub-Saharan Africa; births by teenage mothers have been reported to account for the highest infant and child mortality. In 2007, the under-five mortality rate among children born to adolescent mothers in Ghana was 124/1000 and this was significantly higher than the national rate of 82/1000 [9]. Young mothers are more likely to experience complications during pregnancy and delivery than older mothers [8, 9]. Adolescent pregnancy exposes young women to medical, social and economic risks. They have a high risk of dying during childbirth and of being socially excluded by the society such as by family members, teachers and peers. These factors may expose female adolescents, especially single mothers, to poverty [6]. Urban centres are growing fast in the global South and majority of the youth will be living in urban areas in the next decade [10]. However, very little is known about adolescent sexual and reproductive health in the urban context [11].

Studies on sexual and reproductive health of adolescents tend to focus on vulnerability and exposure to various health and developmental risks, including teenage pregnancy [4–6, 10–12]. In the midst of these risks and vulnerabilities, some adolescent girls are able to either avoid teenage pregnancy or cope well with it. This requires investigation to understand how they acquire competencies in avoiding or dealing with teenage pregnancy [11].

Research on resilience (adaptive capacity) has a long history among Western psychologists and social workers, mostly in the United States [13, 14]. In recent times, however, psychologists have started conducting research in low and middle income countries [15, 16]. Obrist and colleagues conceptualized resilience from a social science perspective leading to the development of the multi-layered social resilience framework [17]. This framework puts emphasis on the human capacity to act in response to a threat by defining social resilience as “the capacity of actors to access capitals in order not to only cope with and adjust to adverse conditions (reactive capacity) but search for and create options (proactive capacity), and thus develop increased competence (positive outcomes) in dealing with a threat” [17]. Access to economic, social and cultural capitals is to a large extent structured by power-related symbolic capital [17–19].

While it is acknowledged that sexual and reproductive health research should involve males and females, this paper focuses on adolescent girls aged 15 to 19 years only. This age group was selected because, they are more likely to get pregnant than the younger adolescents (aged 10 to 14 years). The paper examined how actors (family, peers etc.), institutions and organizations (schools, health services etc.) influence adolescent girls’ competencies in preventing or dealing with teenage pregnancy. The study is embedded in a larger mixed methods project on reproductive resilience that was implemented in urban as well as rural research sites in Tanzania and Ghana. This paper reports only quantitative findings from the urban site Accra, Ghana. Quantitative findings from urban Tanzania will be reported elsewhere.

## Research method

### Study site

The study was conducted in the Accra Metropolitan Assembly (AMA) in the Greater Accra region of Ghana. Accra Metropolitan Assembly (AMA) is the political, economic and administrative capital of Ghana with a population of 1,848,614 in 2010 [20]. The Greater Accra region is the most densely populated region in the country with AMA being the most densely populated part of that region. AMA is made up of eleven sub-Metropolitan District Councils namely; Ablekuma Central, Ablekuma North, Ablekuma South, Ashiedu Keteke, Ayawaso Central, Ayawaso East, Ayawaso West, La, Okaikoi North, Okaikoi South and Osu Klotey with a total land area of 173 square kilometres.

Each of the 11 sub-metros is treated more or less as an administrative district and each of the sub-metros is served by a government polyclinic and several small government and private clinics. There are four government hospitals, five quasi-government hospitals and one teaching hospital in the Accra metropolis.

### Study population and sampling

Three out of the 11 sub-metros were randomly selected. These were: Ashiedu Keteke; La and Okaikoi North with a total population of 117,525; 96,790 and 117,590 respectively. The study applied a cross-sectional survey approach focusing on female adolescents aged 15–19 years. A total of 820 young girls were interviewed. Out of them, 692 were never pregnant girls and 128 were pregnant girls or young mothers. A minimum sample size of 775 was calculated for the study [21]. To attain the required sample size for the study, taking into account the adolescent population and teenage pregnancy rate in Accra [20], 18 enumeration areas (EAs) (six in each sub-metropolis) were selected. Every residential dwelling in the 18 EAs was visited to identify respondents for the study. At the end of fieldwork, only four girls refused participation, mainly because their caretakers refused to give consent.

### Data collection

Data collection was done from December 2010 to February 2011 by females aged 16 to 22 years using a structured questionnaire with pre-coded possible responses. A peer-to-peer data collection approach was deliberately adopted to remove any age-related social barriers, to enhance rapport and to create mutual trust between the interviewees and interviewers. Field reports indicated that respondents saw interviewers as their colleagues and therefore opened up to them; some became friends and even exchanged their mobile phone numbers. Field workers were thoroughly trained and were supervised during data collection. The data collection tools were pre-tested outside the study sub-metropolis to ensure clarity, consistency and reliability. Findings and experience gained from the pre-testing were used to revise the tool before the commencement of the main data collection.

### Sexual and reproductive resilience

Since resilience must be built against a threat, this study was guided by the Multi-layered Social Resilience framework [17], which was adapted to the context of teenage pregnancy. The framework helped to conceptualized possible factors that could influence adolescents’ competence to either prevent or cope with teenage pregnancy. Building on the assumption from the public health literature that unwanted teenage pregnancy is a potential threat to young girls. It has been widely reported that teenage pregnancy is a threat to the health, education, social and economic wellbeing of adolescent girls [5, 22].

**Fig. 1:**
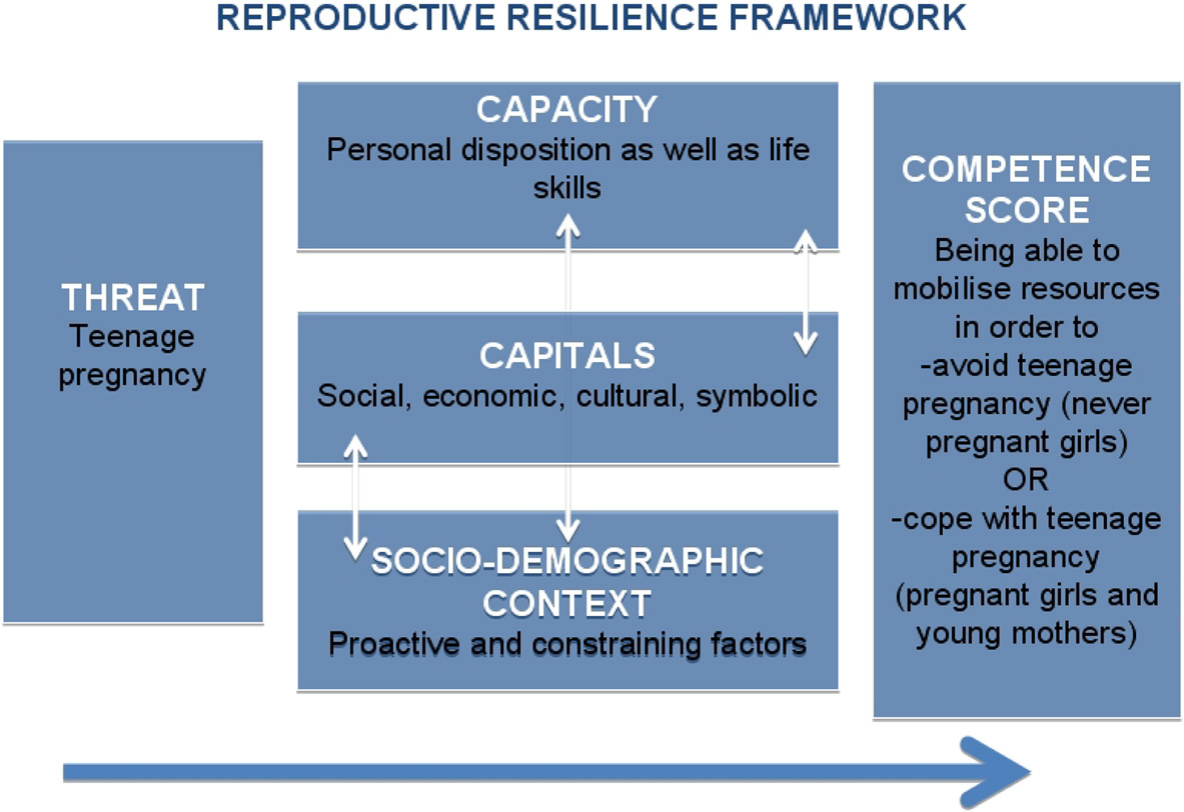
Reproductive Resilience framework (modified Multi-layered Social Resilience framework by Obrist et al., 2010). The frame work illustrates how a threat of teenage pregnancy could be influenced by the capacity- personal disposition and life skills; Capitals- social, economic, cultural and symbolic and Socio-demographic context which could enhance proactive actions or becomes constraining factors. As indicated by the arrows, these factors interact with each other to produce competence levels of adolescents to develop resilience against teenage pregnancy of cope well with it when it occurs

Drawing on the Multi-layered Social Resilience framework [17], we examined the building of resilience against the threat of teenage pregnancy at the household level. We emphasized on: 1) social, economic, cultural and symbolic capitals of female adolescents; 2) their capacities; 3) their socio-demographic context, and 4) the outcome, which is the ability to competently deal with the threat of teenage pregnancy (Fig. 1). Thus, two sets of questionnaires were designed to address similar but specific issues of pregnant girls/young mothers on one hand and never pregnant girls on the other hand. In this study, we defined pregnant girls/young mothers to include all those who had ever been pregnant (were pregnant at the time of the study, had given birth and self reported miscarriage or abortion). The questions allowed for comparison between the two groups of respondents.

Building on the multi-layered social resilience framework, ‘competence’ was adopted as a proxy for resilience. Competence in this study involves the relationship between capacities, capitals and the socio-demographic characteristics that enable young girls to reactively cope with pregnancy or proactively prevent pregnancy. As a measure of social resilience, the competence score was computed based on the following outcomes: being able to competently mobilize resources in order to prevent teenage pregnancy (never pregnant girls) or cope well with teenage pregnancy (pregnant girls/young mothers).

### Data analysis

Data analysis was done using EpiInfo version 3.2 software to perform descriptive statistics, chi-square and bivariate and multivariate logistic regression. A cumulative competence scale was developed in order to gain insights about reproductive resilience. In both sites, Tanzania and Ghana, the same cumulative score based on 10 competence indicators that refer to the reactive and proactive mobilization of resources was computed. Depending on the pregnancy status (never pregnant girls or pregnant girls/young mother) a set of questions related to competencies were developed for the study (Table 1). Each competence-question answered with “yes” (i.e. having actively mobilized resources, continued education) contributed ‘1’, questions answered with “no” (i.e. having not actively mobilized resources, did not continue with education) contributed “0” to the score. Among the pregnant girls and/or young mothers each respondent could score the minimum of 0 (all questions answered with “no”) and the maximum of 10 (100 %). For the purpose of this analysis the score of ≤50 % was determined as ‘low competence in mobilizing resources to avoid or deal with pregnancy’ and the score of 51–100 % as ‘high competence in mobilizing resources to avoid/deal with pregnancy’. A 50 % cut-off point was used in order to learn about the broad spectrum of competencies among respondents. We computed bivariate relationship between competence score and capital variables; competence score and ability variables; competence score and demographic variables. Variables for consideration in logistic models to identify determinants of resilience were identified by suggestive bivariate relationships (*p* ≤ .20). All logistic regression models were control for age, since we know that proportionally more never pregnant girls were in the younger age than the pregnant/young mothers (Table 2). The outcome variable of the study is the ability to avoid pregnancy or cope well with it, which is expressed in the competence scores. Therefore the hypothesis for the logistic regression model was that “a high resilience score is associated with the social, economic, cultural and symbolic capitals as well as life skills and capacities”.

**Table 1:** Adolescents reproductive resilience research design

Variables	Questions
1. Socio-demographic context: *Age, location, level of education, religion, wealth of family, family status, relationship status*	How old are you? What is your educational level? Are you in a relationship? Is your father living together with your mother? Have you ever had sex? What is your religion? Is your father having another wife beside your mother? What is your ethnicity? What is your family’s source of income? Have you ever been pregnant?
2. Capitals:	
2.1 Social capital; *Various kinds of valued relations with significant others*	Do you have someone you can turn to in case you have questions related to avoiding/dealing with teenage pregnancy? How many people can you turn to? Whom do you turn to?
2.2. Cultural capital; *Embodied (personal dispositions and habits), objectified (knowledge and tradition stored in material forms) and institutionalized (educational qualification)*	Do you have access to other information sources in order to learn about how to avoid/deal with teenage pregnancy? How many different sources do you have access to? What kind of sources?
2.3. Economic capital; *Command over economic resources, mainly cash and assets*	Do you have someone you can turn to in case you need money to avoid/deal with teenage pregnancy? How many people can you turn to? Whom do you turn to?
2.4. Symbolic capital; *Honour, recognition and prestige*	Do you feel accepted within your social environment? Do you strive for a good reputation?
3. Capacities:	
3.1 Psycho-social resilience; *Psycho-social dispositions*	Do spiritual & religious beliefs help you to abstain from sexual relationships/to deal with teenage pregnancy? Do you believe that you can successfully manage to avoid/deal with teenage pregnancy? Do you have the ability to establish and maintain relationships with people, who you can ask for advice related to avoiding/dealing with pregnancy?
3.2 Life skills; *Sexual and reproductive health knowledge*	Do you dare to speak out when someone approaches you in a sexual way and you do not want it? Do you know how to protect yourself from pregnancy? Do you decide freely if, when and with whom you want to have sex?
4.1 Competencies of never pregnant girls; *Being able to competently mobilize resources in order to avoid teenage pregnancy*.	Do you protect yourself from pregnancy by using contraceptives? Did you deliberately abstain from sex to avoid pregnancy? Have you mobilized any social support to actively avoid pregnancy? Did you manage to mobilize this support successfully? Have you actively mobilize economic resources to protect yourself from pregnancy? Did you manage to mobilize them successfully? Have you actively looked for other sources of information on how to protect yourself against pregnancy? Did you manage to get the information you were looking for? Do you dare to speak out when someone approaches you in a sexual way that you do not like? Do you decide freely when and with whom to have sex?
4.2 Competencies of pregnant girls/young mothers; *Being able to competently mobilize resources in order to cope with teenage pregnancy/childbirth*.	Did you try to continue schooling or learn a trade during/after pregnancy? Did you make use of health services to check your own health and/or the health of your baby? Have you actively mobilized any social support in order to cope with teenage pregnancy/childbirth? Did you manage to mobilize this support successfully? Have you mobilized any financial resources in order to cope with teenage pregnancy/childbirth? Did you manage to mobilize these resources successfully? Have you actively looked for other sources of information on how to cope with teenage pregnancy/childbirth? Did you manage to get the information you were looking for? Do you dare to speak out when someone approaches you in a sexual way that you do not like? Do you decide freely when and with whom to have sex?

**Table 2:** Demographic characteristic of respondents by pregnancy status

Variables	Never pregnant Frequency (%) *n* = 692	Pregnant girls/young mothers. Frequency (%) *n* = 128	X^2^ (P value)
Age (years)			66.04 (<0.001)
15	166 (24.0)	5 (3.9)	
16	97 (14.0)	7 (5.5)	
17	100 (14.5)	16 (12.5)	
18	186 (26.9)	35 (27.3	
19	143 (20.7)	65 (50.8)	
Relationship status			63.13 (<0.001)
Single	456 (65.9)	28 (21.9)	
Married	6 (0.9)	26 (20.3)	
In relationship but not married	230 (33.2)	74 (57.8)	
Education			63.43 (<0.001)
Primary education	55 (7.9)	22 (17.2)	
Junior high school	302 (43.6)	56 (43.7)	
Senior high school & above	264 (38.1)	23 (18.0)	
Others (non-formal education, vocational training etc.)	71 (10.2)	27 (21.1)	
Both parents staying together			10.41 (0.005)
Yes	352 (50.9)	47 (36.7)	
No	316 (45.7)	72 (56.3)	
Others (dead, don’t know father)	24 (3.5)	9 (7.0)	
Father has more than one wife			0.23 (0.726)
Yes	254 (36.7)	55 (43.0)	
No	438 (62.3)	73 (57.0)	
Others (dead, don’t know^a^ father)	9 (1.3)	2 (1.6)	
Religion			4.77 (0.092)
Christian	535 (77.3)	100 (78.1)	
Muslims	96 (13.9)	7 (5.5)	
Other	61 (8.8)	21 (16.4)	

^a^was excluded from the Chi-square analysis as values are too small but could neither be added to ‘Yes’ or ‘No’ responses

In doing so, we looked at: Social capital, which is defined as all kinds of relationships that are cherished by those involved [17, 23]. In this study, access to social capital referred to the ability to turn to these relations for advice and social support on how to either avoid or cope with teenage pregnancy and Economic capital, which in this study refers to having access to economic resources, either in cash or kind [17, 23]. Thus, adolescent girls should have access to resources that they could make use of to either prevent or cope with pregnancy. Finally, the study built on the definition of cultural capital as personal habits and dispositions, knowledge, traditions and educational qualifications that are available for a person to draw upon when needed [17, 23]. In the context of teenage pregnancy it meant that adolescent girls should have access to and be able to mobilize different media resources to either prevent or cope with pregnancy. Findings were compared between the two groups (never pregnant girls and pregnant girls/young mothers) where appropriate.

### Ethical considerations

The study was approved by the Institutional Review Board of the Noguchi Memorial Institute for Medical Research, University of Ghana, Ghana (clearance number 052/08-09). A written informed consent was collected from each respondent either directly from those aged 18 years and above or from the guardians (mainly parents) of those less than 18 years. In addition, oral informed consent was collected from each respondent at the point of interview. Confidentiality of information given was assured and individuals were given the freedom to withdraw their participation at any point in the interview if they so wish. Participants were informed about their right to refuse to answer any question that they felt uncomfortable with in the course of the interview.

## Results

### Socio-demographic characteristics

Out of 820 adolescents aged 15–19 years interviewed, 128 (16 %) were pregnant or already mothers. The socio-demographic characteristics of the respondents are presented in Table 2. Significantly more never pregnant girls were younger than the pregnant girls or young mothers. On the other hand, the age of 19 years was significantly related to being pregnant/young mother (*P* < 0.0001). Significantly more pregnant girls/young mothers terminated their schooling at primary school level compared to never pregnant girls. Most of the never pregnant girls were still in school. More (*P* = 0.0043) never pregnant girls (50 %) had their parents (mother and father) living together compared to pregnant girls/young mothers (37 %). Thus, those whose parents were living together were less likely to start childbearing. Most socio-demographic variables did not influence the competence scores of respondents in both groups. However, chi-square analysis showed a significant relationship between education and competence (*P* = 0.0133) to avoid teenage pregnancy, suggesting that adolescents with more years of schooling were more likely to develop competence to avoid teenage pregnancy.

### Competence

A slight majority of respondents in both never pregnant (51 %) and pregnant girls/young mothers (60 %) groups had high competence scores. The mean (±SD) competency scores were 5.4 (2.8) and 6.2 (2.7) among never pregnant girls and pregnant girls/young mothers respectively. The median scores were 6.0 and 6.5 among never pregnant girls and pregnant girls/young mothers respectively with a range of 0 to 10 in both groups. Most pregnant girls/young mothers reportedly knew how to deal with health problems that affect their own health and that of their babies and maintained that they have the ability to mobilize information and support needed to do so. Never pregnant girls with high competence scores were able to avoid pregnancy because they could mobilize information and support to do so.

### Access to social capital

About the same proportion of adolescents in both groups, never pregnant (62 %) and pregnant girls and young mothers (68 %) had access to social capital. There was a significant relationship between social capital and competence in both groups (never pregnant: X^2^ = 91.349, *P* < 0.001) and (pregnant girls and young mothers: X^2^ = 4.078, *P* = 0.043). Depending on the pregnancy status, adolescents turned to different people. Prominent social actors consulted for advice were parents, other relatives and husbands/partners (Table 3).

**Table 3:** Individuals that adolescents in Accra turn to for support by pregnancy status^a,b^

Whom to turn to for support	Never pregnant; frequency (%) *n* = 692		Pregnant girls and young mothers; frequency (%) *n* = 128	
	Social support	Economic support	Social support	Economic support
Parents	459 (66.3)	457 (66.0)	66 (51.6)	52 (40.6)
Other relatives	182 (26.3)	182 (26.3)	53 (41.4)	22 (16.8)
Husband/partner	172 (24.8)	172 (24.8)	37 (28.9)	91 (71.3)
Peers	80 (11.6)	77 (11.1)	28 (21.9)	6 (4.7)
Teachers	40 (5.8)	39 (5.6)	1 (0.8)	0
Religious leaders	37 (5.3)	37 (5.3)	3 (2.3)	0
Nurses/doctors	13 (1.9)	13 (1.9)	19 (14.9)	1 (0.8)

^a^Sorted in column 2 in descending order

^b^Multiple choices were allowed

Multiple logistic regression analysis showed that only parents, as a cherished relation, contributed significantly to competence building among never pregnant girls (Odd ratio 1.4998; *p* = 0.050). In addition, pregnant girls and young mothers who consulted nurses or doctors were more able to cope well with pregnancy and childbirth (Odd ratio 7.1912; *P* = 0.005) (Table 4).

**Table 4:** Multiple Logistic Regression models for capitals and competence score (dependent variable) among adolescent girls by pregnancy status^a*^

Social & cultural capital variables	Never pregnant girls				Pregnant and young mothers			
	Odds Ratio	95 %	C.I.	*P*-Value	Odds Ratio	95 %	C.I.	*P*-Value
Model 1: Social capital								
Husband/partner	0.6407	0.2759	1.4880	0.300	2.9272	0.9661	8.8689	0.058
Nurses/doctors	1.4348	0.3302	6.2341	0.630	7.1912	1.8068	28.6216	0.005
Other relatives	1.0414	0.6947	1.5611	0.844	1.4532	0.4938	4.2767	0.497
Parents	1.4998	0.9990	2.2516	0.050	0.7479	0.2539	2.2035	0.598
Peers	0.8350	0.5398	1.2918	0.418	2.4893	0.7362	8.4176	0.142
Religious leaders					4.1876	0.1996	87.8472	0.356
Teachers	1.4145	0.8552	2.3397	0.177				
Model 2: Cultural capital								
Books	1.4293	0.0168	2.0091	0.039	1.7165	0.4608	6.3945	0.420
Brochure	0.3134	0.1341	0.7321	0.007	1.0675	0.0313	36.4024	0.971
Cell Phones					0.4231	0.0891	2.0104	0.279
Magazines					1.9136	0.3187	11.4897	0.478
Music songs	0.6170	0.3582	1.0631	0.082	5.0131	1.0565	23.7879	0.042
Radio					0.9022	0.3455	2.3555	0.833
Television (TV)	1.9244	1.1769	3.1466	0.009	1.4982	0.4065	5.5218	0.544

^a^Sorted in column 1 in alphabetical order under the headings “Social capital” and “Cultural capital”

*Age and marital status were included in the model as control variables

### Access to cultural capital

There was a significant relationship between access to the media and competence score in both groups (never pregnant: X^2^ = 9.060, *P* = 0.003; and pregnant girls and young mothers: X^2^ = 5.231, *P* = 0.022). Prominent sources of information consulted in both groups were Television, Radio and Books (Table 5). However, a multiple logistic regression analysis showed that access to TV (Odd ratio 1.9244; *P* = 0.009) and books (Odd ratio 1.4293; *P* = 0.039) were significantly related to high competence score while information brochure (Odd ratio 0.3134; *P* = 0.007) was negatively related to competence score among the never pregnant girls. On the other hand, only music songs was significantly related to competence score among the pregnant girls/young mothers (Odd ratio 5.0131; *P* = 0.042) (Table 4).

**Table 5:** Sources of information used by adolescent girls in Accra by pregnancy status ^a, b^

Cultural capital	Never pregnant frequency (%) *n* = 692	Pregnant girls and young mothers frequency (%) *n* = 128
TV	583 (84.2)	109 (85.1)
Radio	421 (60.9)	86 (67.3)
Books	338 (48.9)	19 (14.9)
Magazines	107 (15.4)	8 (5.9)
Music songs	84 (12.1)	11 (8.9)
Cell Phones	56 (8.1)	18 (13.9)
Brochures	44 (6.3)	3 (2.0)
Billboards/posters	28 (4.0)	4 (3.0)
Other	28 (4.1)	4 (3.0)

^a^ Sorted in column 2 in descending order

^b^ Multiple choices were allowed

### Access to Economic capital (Ability to organize economic support)

Having access to economic capital had a significant relationship with the competence score among never pregnant girls (X^2^ = 15.76, *P* < 0.001) but not for the pregnant girls and young mothers (X^2^ = 3.280, *P* = 0.070), 79 % of the pregnant girls and young mothers compared to 38 % of the never pregnant girls had access to economic capital. Pregnancy status may determine whom adolescent girls turn to for financial assistance either to prevent pregnancy or care for their pregnancy and baby. However, a multiple regression analysis (Table 6) showed that access to economic capital contributed to high competence scores in both groups, never pregnant girls (Odd ratio 28.0000; *P* < 0.001) and pregnant girls/young mothers (Odd ratio 11.4290; *P* < 0.001).

**Table 6:** Multiple Logistic Regression analysis of the relationship between selected abilities and competence score (dependent variable) among adolescent girls in Accra^a*^

Capacity variable	Never pregnant girls’ competence score				Pregnant and young mothers’ competence score			
	Odds ratio	95 %	C.I.	*P*-value	Odds ratio	95 %	C.I.	*P*-value
Ability to establish relationship with others	2.7133	1.6085	4.5769	<0.001	0.8172	0.2679	2.4928	0.723
Ability to organize economic support	28.000	13.4281	58.3854	<0.001	11.4290	4.4061	29.6463	<0.001
Dare to speak against sexual advances	1.4025	0.7247	2.7143	0.315	0.9899	0.1675	5.8500	0.991
Decide with whom and when to have sex	1.0761	0.7007	1.6524	0.738	0.5922	0.2023	1.7332	0.339
Deliberately abstain from sex to prevent pregnancy	2.4420	1.5074	3.9562	<0.001				
Have sexual and reproductive right	1.6570	1.0927	2.5128	<0.001	2.8125	0.9891	7.9977	0.052
Make use of health services to protect own and baby’s health					7.3926	1.9406	28.1619	<0.001
Religious beliefs help to prevent pregnancy	1.5876	0.9717	2.5943	0.065				
Use contraceptives to prevent pregnancy	7.1334	4.4571	11.4165	<0.001				

^a^Sorted in column 1 in alphabetical order

*Age and marital status were included in the model as control variables

### Symbolic capital among adolescent girls

Symbolic capital such as *striving for a good reputation in relation to sexual behaviour* and *feeling accepted within their social environment* was examined. Both never pregnant girls and pregnant girls/young mothers attached high importance to these symbolic capitals. When asked, whether they *actively strive for good reputation in their communities in terms of sexual behaviour,* 78 % of never pregnant girls and 76 % of pregnant girls/young mothers responded in the affirmative. The young study participants maintained that they consciously behave in culturally and socially acceptable ways in order to avoid being labelled as ‘bad girls’ in the community. In addition, 83 % of the never pregnant girls and 81 % of the pregnant girls/young mothers said they *feel accepted within their social environment*. Thus, pregnancy status did not affect their positions in the social environment where they lived as there was no difference between the two groups.

### Relationship between selected capacities and competence score

Capitals available to adolescent girls may shape their capacities such as the ability to mobilize social support to either prevent or cope well with pregnancy. A multiple logistic regression analysis showed that some of these capacities contributed to a high competence score among respondents. Capacities such as “the ability to organize economic support (access to economic capital) when needed (Odd ratio 28.0000; *P* < 0.001)”, “the ability to use contraceptives to prevent pregnancy (Odd ratio 3.0804; *P* < 0.001)” and “deliberately abstaining from sex to prevent teenage pregnancy (Odd ratio 2.4420; *P* < 0.001)” among others were strongly related to competence score among the never pregnant girls. Also, among pregnant girls/young mothers, capacities such as “the ability to organize economic support (access to economic capital) when needed (Odd ratio 11.4290; *P* < 0.001)” and “the ability to make use of health services to protect own and baby’s health (Odd ratio 7.3926; *P* = 0.003)” were significantly related to the competence score (Table 6).

## Discussion

This paper utilized a reproductive resilience research design that built on the Multi-layered Social Resilience framework [17], to establish relationships between social, economic and cultural capitals as well as contextual factors that enhance the competence of adolescent girls to either proactively prevent or reactively cope well with pregnancy and child birth in Accra. In this paper, competence was measured as a proxy for resilience, which is viewed as the process of, and capacity for, adapting successfully to challenging or threatening circumstances, or the ability to resist risk and overcome adversity.

Findings that older and less educated girls were more likely to start childbearing compared favourably with what was reported by the Ghana Demographic Health Survey in 2008, where adolescent’s chances of childbearing increases with age and reduces with education [24]. Compared to 2008 [24], teenage pregnancy among older adolescents in Accra rose from 11 % to 16 %, an increase of about 5 %. However, most pregnant girls and young mothers in our study defied the general view of them as vulnerable people [25–27]. Instead they developed skills and competencies to cope well with pregnancy and childbirth [28]. It has been severally established in resilience literature that major life transitions can provide opportunities for developing resilience [29–31] and this has been confirmed in our study. What might be regarded as a threat in one situation could become a protective factor in another situation [18]. This finding contradicts the generally held view that adolescent mothers are vulnerable children who are having children at a time that they are not supposed to [4–6, 18, 32]. It must be acknowledged that teenage pregnancy could have a negative effect on adolescents as it may truncate their education and other social and economic developments [33]. However, findings reported here suggest that this might have been over exaggerated and stated out of context. Obrist and Mlangwa [34] argued that teenage pregnancy must be seen in the context within which it occurs, thus it should not be de-contextualized and labelled as a health problem.

The finding that parents were the single most prominent social actors consulted by adolescent girls for sexuality related information is an emerging trend in parenting in Ghana. It emphasizes the critical role of parents in providing sexual education to their adolescent daughters. This is changing the relationship between parents and their children. In the Ghanaian context, relatives such as aunts and grandmothers are expected to provide sexuality education and other sexual and reproductive supports to their nieces and grandchildren. However, due to societal changes, where individuals have moved away from their ancestral homes to work in urban areas with weak or severed kinship ties, the parents and most importantly mothers are being consulted on sexual matters [35, 36]. It has been argued that parent–child communication on sexual matters tend to be authoritarian and vague with parents often overwhelmed with their new roles as they do not know how to provide sexual education and instead of promoting healthy and meaningful discussions, children are often left more confused [35] and in their attempts to clear this confusion, they may fall victim to information sources of varying quality. There is therefore a need to enhance the communication skills of parents, especially mothers to communicate effectively with their teenage girls on sexual matters.

As the changing family structure confers the role of sexual educators on parents, especially mothers, who are often not able to fill the information gap for their adolescent children, access to other information sources like the mass media has become very important for the youth to fill that gap [36]. It was not surprising that TV contributed significantly to competence among never pregnant girls but not pregnant girls/young mothers because mass media campaigns often do not target the latter. It is argued that mass media should also focus on this neglected group to enhance their competence to deal with pregnancy and child rearing.

The finding that fewer never pregnant girls were seeking for economic support to deal with sexual and reproductive health related issues, compared to pregnant girls and young mothers, could be attributed to the fact that, it is not culturally acceptable for adolescent girls to ask for financial support for pregnancy prevention measures like contraceptives. Doing so may attract the label of being a ‘bad girl’. However, it must be recognized that at any given time, a good number of teenagers will be sexually active and therefore exposed to all kinds of risks associated with engaging in ‘unsafe sex’ including pregnancy and sexually transmitted diseases like HIV/AIDS [28, 37, 38]. It is therefore in the interest of society to encourage and support adolescents, especially girls to develop skills and the capacity to be able to negotiate for safe sex and be able to decide on when, and who, to have sex with.

Over 70 % of the never pregnant girls said they knew how to prevent pregnancy, however, it has been reported in several studies from Ghana that being knowledgeable about contraceptives and birth control in general does not always translate into actual protection [9, 24, 27, 37]. As such, there is a need to use this high level of knowledge on contraceptives to design interventions that will empower and support these girls to develop capacity to actually use contraceptives and other birth control methods to prevent unwanted pregnancy. In this study it was found that about 50 % of adolescent mothers returned to school or have started various apprenticeships to learn one trade or the other and this was good, however, additional interventions are required to enhance the capacity of the remaining half of these girls to return to school or enter apprenticeship after childbirth. In Ghana, the policy is to encourage teenage mothers to go back to school or enter into apprenticeship and this policy must be enforced in a manner that goes beyond encouragement but also build capacities of teenage mothers to return to school especially or enter into apprenticeship. However, it has to be recognized that some of the adolescent mothers will marry, which may affect their capacity to return to school or enter into apprenticeship, yet there should be a system to help married adolescents acquire some skills that will enable them to earn decent living and be able to support themselves and their children in one way or the other.

The adaptation of the Multi-layered Social Resilience framework [17], to study adolescent sexual and reproductive health with emphasis on teenage pregnancy clearly demonstrated that resilience is not a static situation that must be built once for all threats, rather it is a process that is influenced by a given threat within a prevailing context.

### Weakness of the study

The weakness of the study is acknowledged in the fact that the quantitative approach used did not cover local constructions of categories mentioned by adolescent girls themselves. Also, resilience is a dynamic phenomenon that is developed against a given threat within a given context. So a longitudinal study that follows adolescents’ overtime, preferably from age 10 to 19 years, may provide a better understanding of the resilience of adolescent girls against teenage pregnancy. A cross sectional study can only generate limited information to help understand the complexity of social interactions within which adolescents experience sexuality. Also, the exclusion of adolescent boys from the study is a weakness that has been recognized and being addressed in a follow-up qualitative study designed to address local representations of capitals, capacities and competences, which will be elaborated elsewhere.

## Conclusion

Adolescent girls, especially those that get pregnant should not be viewed as weak and vulnerable because many of them in our study have developed competencies to cope with pregnancy and childbirth effectively. However, there is a need for sexual and reproductive health promotion, for example, through the use of mass media, to target both never pregnant and pregnant adolescents and young mothers to provide them with relevant information on how to go through pregnancy and childbirth. Focusing on developing competencies of girls to access social, economic and cultural capitals may be a more effective way of tackling the threat of teenage pregnancy than focusing only on their vulnerabilities and risks associated with it.

This paper, using the reproductive resilience framework examined how various material and non-material resources contribute to the competence building of adolescents to either proactively avoid teenage pregnancy or to reactively cope well with it. In the context of our study, and for that matter, sub-Saharan Africa, adolescent pregnancy in itself may not present a health risk but the lack of social and economic supports for the girls to cope with teenage pregnancy may be a bigger risk for these girls [39]. Like other studies [40], we suggest that adolescent girls should be looked at as active social agents who are not only shaped by the environment around them but who also actively shape their environments and structures that they interact with daily at the household and community levels and in the process develop resilience to deal with some of the challenges that they face.
